# High-Sensitivity Troponin I and Cardiovascular Events in Stable Coronary Artery Disease: Insights from a Longitudinal Outpatient Study

**DOI:** 10.3390/ijms242417286

**Published:** 2023-12-09

**Authors:** Celia Maria Cassaro Strunz, Whady Hueb, Paulo Cury Rezende, Sabrina Pacheco do Amaral Vendramini, Arthur Cicupira Rodrigues de Assis, Alessandra Roggerio, Maria Stanislavovna Tairova, Marcela Francisca Silva, Senili Avila Oliveira, Gyovanna de Cassia Agreste Kisser, Roberto Kalil Filho

**Affiliations:** 1 Clinical Laboratory, Instituto do Coracao (InCor), Hospital das Clinicas HCFMUSP, Faculdade de Medicina, Universidade de Sao Paulo, Sao Paulo 05403-900, SP, Brazil; sabrina.vendramini@incor.usp.br (S.P.d.A.V.); alessandra.roggerio@incor.usp.br (A.R.); senili.avila@hc.fm.usp.br (S.A.O.); gyovanna.kisser@hc.fm.usp.br (G.d.C.A.K.); 2 Clinical Division, Instituto do Coracao (InCor), Hospital das Clinicas HCFMUSP, Faculdade de Medicina, Universidade de Sao Paulo, Sao Paulo 05403-900, SP, Brazil; whady.hueb@incor.usp.br (W.H.); rezendepaulo@hotmail.com (P.C.R.); arthur.cicupira@hc.fm.usp.br (A.C.R.d.A.); mariatairova@hotmail.com (M.S.T.); marcela.silva@hc.fm.usp.br (M.F.S.); kalil@uol.com.br (R.K.F.)

**Keywords:** high-sensitivity troponin I, cTnI/99th tertiles, stable coronary artery disease, cardiovascular events

## Abstract

Numerous studies have been published suggesting that troponin levels are related to adverse outcomes in chronic cardiac and non-cardiac conditions. Our study investigated whether troponin levels gathered from unselected blood samples taken during outpatient care are associated with adverse outcomes in a population with stable coronary artery disease. In a cohort of 949 patients with stable coronary artery disease, an average age of 67.5 ± 9.5 years, 69.5% male, 52.1% diabetics, 51.6% with previous myocardial infarction, and 57.9% with triple-vessel disease, 21.7% of patients encountered new events during an average period of monitoring of 2.07 ± 0.81 years. Troponin I/99th percentile categorized into tertiles emerged as an independent predictor of death and combined events risk (hazard ratio: 2.02 (1.13–3.60), *p* = 0.017; 2.30 (1.37–3.88, *p* = 0.002, respectively). A troponin ratio > 0.24 was able to identify 53.3% of patients at risk of death and heart failure hospitalization. In patients with stable coronary artery disease who are adherent to treatment, troponin levels are independently associated with death and heart failure hospitalization in a medium-term follow-up.

## 1. Introduction

Cardiac troponin (cTn), a structural protein found in the contractile apparatus of cardiac muscle, is the preferred biomarker for detecting myocardial necrosis. In 2018, the ESC/ACCF/AHA/WHF guidelines introduced the term “high sensitivity cardiac troponin” (hs-cTn) to describe newly available assays. These assays can detect troponin levels in more than 50% of healthy individuals [1] and have proven effective in both diagnosing acute myocardial infarction (MI) and predicting patient outcomes [2,3,4].

Elevated troponin levels have been observed in various clinical conditions, including non-ischemic cardiac conditions like stable coronary artery disease, heart failure (HF), arrhythmias, and non-cardiac conditions such as stroke, sepsis, thromboembolic events, oxidative stress, and intense physical activity [5,6]. Several studies have suggested that the chronic elevation of troponin in patients with cardiac damage but without necrosis can serve as a prognostic factor for adverse outcomes, such as MI, HF, and cardiovascular and all-cause mortality [7,8,9].

A meta-analysis of 28 studies involving 154,052 individuals without clinical manifestations of myocardial injury indicated that troponin within the normal range is associated with an increased risk of cardiovascular disease (CVD) [10]. However, another study conducted on a group of 20,000 patients found that the 99th percentile cutoff value for troponin in outpatients differed from the value indicated by the manufacturer. This suggests that troponin levels should be interpreted cautiously in stable populations [11].

Considering that troponin levels can be elevated due to cellular damage not caused by acute illness and that undisclosed chronic conditions may contribute to the release of this biomarker, it is crucial to investigate the role of troponin in identifying various clinical conditions.

The aim of this study was to determine whether troponin levels gathered from unselected blood samples taken during outpatient care are associated with adverse events in a population with stable coronary artery disease.

## 2. Results

Of the 1003 patients initially assessed for the study, 32 did not have coronary artery disease (CAD), 15 had chronic kidney disease, and 7 had incomplete clinical data, resulting in a final study cohort of 949 patients. They were monitored for an average period of 2.07 ± 0.81 years.

The patients had a median age of 67.5 years, and 69.5% of them were male. This group of patients was predominantly composed of individuals who had diabetes (52%), hypertension (71.9%), and dyslipidemia (75.4%). Additionally, 526 patients (57.9%) had triple-vessel disease, and 486 (51.6%) had a history of a prior MI, which had been managed with either percutaneous coronary intervention (PCI) (40%) and/or coronary artery bypass grafting (CABG) (35%).

The clinical characteristics and laboratory results can be found in Table 1.

Troponin levels exceeding the detection limit of 2.5 ng/dL were observed in 95% of the patients, which was higher than what is specified by the manufacturer for a healthy population (72%). Remarkably, even in the absence of a recent episode of myocardial injury, 9.5% of the patients had troponin levels surpassing the 99th percentile.

Separate analyses on TnI/99th diabetic and non-diabetic patients were carried out. There were no differences between TnI values: non-diabetic TnI/99th, 0.18 (95% CI: 0.17–0.20); diabetic TnI/99th, 0.17 (95% CI: 0.16–0.19), *p* = 0.677.

Patients were categorized into tertiles based on their troponin levels relative to the 99th percentile: group 1 (0.09–0.1 ng/L), group 2 (0.14–0.2 ng/L), and group 3 (0.34–1.12 ng/L). The group with the highest cTn ratio had fewer male participants and a greater incidence of MI, angina, and HF compared to the group with the lowest cTn ratio.

Regarding the other clinical and laboratory variables, no differences were observed among the groups. They all had an equivalent number of patients with diabetes, hypertension, and dyslipidemia, as well as comparable levels of glucose, glycated hemoglobin, urea, creatinine, hemoglobin, HDL cholesterol, LDL cholesterol, and triglycerides. Also, the distribution of the number of vessel diseases was similar among the groups.

Throughout the follow-up period, 206 patients experienced clinical events, constituting 21.7% of the total. The most common event was death, occurring in 88 patients (9.3%), whereas 54.5% died within their first year of participation in the study, followed by PCI/CABG in 62 patients (6.5%), HF hospitalization in 42 patients (4.5%), and MI in 24 patients (2.5%). In a univariate analysis where cTn was stratified into tertiles, the likelihood of death, hospitalization, and combined events was higher in conjunction with the increase in troponin levels (Table 2).

After adjusting for age, sex, previous MI, angina, HF, coronary artery disease, and left ventricular ejection fraction, cTnI/99th percentile ratio treated as a continuous variable emerged as an independent factor associated with higher mortality risk, with an HR (95% CI) of 1.08 (1.03–1.13, *p* = 0.001). Similar results were found when combined events were analyzed, with an HR (95% CI) of 1.07 (1.03–1.11, *p* = 0.0004).

Cox regression was also applied to analyze the association between heart failure hospitalization and risk factors. In a model adjusted by age, HF, and left ventricular ejection fraction, cTnI/99th percentile ratio did not emerge as a significant predictor of hospitalization, with an HR (95% CI) of 1.06 (0.99–1.12, *p* = 0.062).

A higher significant association was observed when Cox analysis was applied in a fully adjusted model considering cTnI/99th percentile categorized into tertiles and using group 1 as a reference. In group 3, for each one-point incremental rise in the cTnI/99th percentile ratio, the risk of mortality and the occurrence of combined events both doubled (Table 3).

In addition to the troponin ratio, age and LVEF were also associated with both death (1.04, 1.02–1.07, *p* = 0.002 and 0.98, 0.96–0.99, *p* = 0.025) and combined events (1.04, 1.01–1.06, *p* = 0.002 and 0.97, 0.96–0.99, *p* = 0.001).

**Figure 1:**
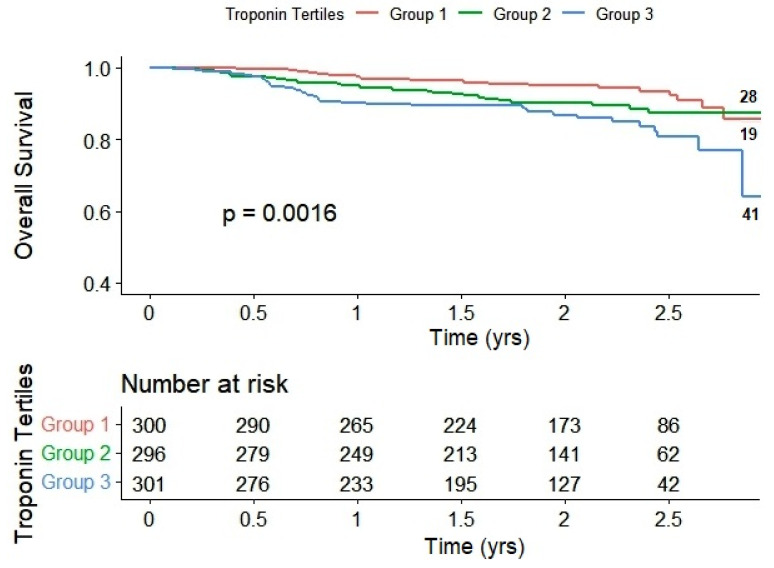
Kaplan–Meier curves of the overall survival of study patients stratified by troponin tertiles.

**Figure 2:**
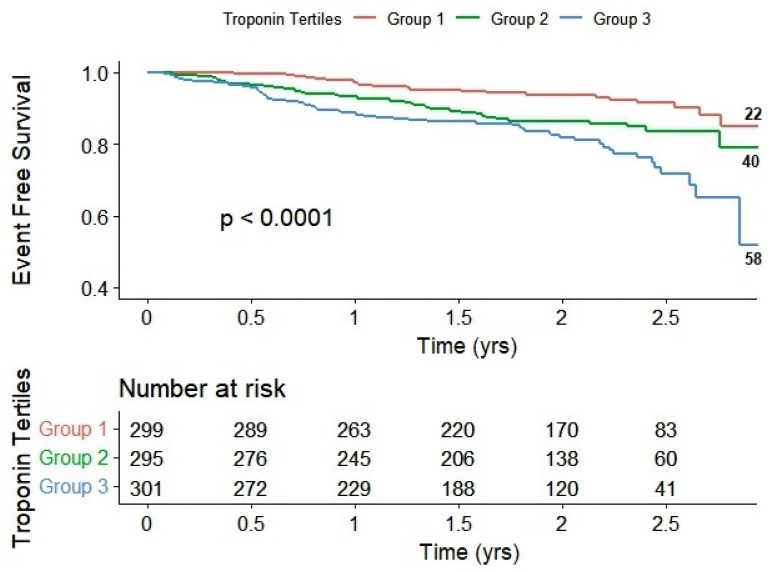
Kaplan–Meier curves of combined event-free survival of study patients stratified by troponin tertiles.

The differences between the three groups become more evident when employing the Kaplan–Meier curve. In comparison to group 1, group 3 exhibited lower survival rates for both mortality and combined events throughout the 2.07 ± 0.81-year study period, as illustrated in Figure 1 and Figure 2, respectively.

*p*-values were calculated considering group 1 as the reference in both models. The number of deaths is indicated on the right side of the graphs.

The optimal cutoff values for troponin concentration and the cTnI/99th percentile ratio were determined by analyzing the area under the curve (AUC), sensitivity, and specificity of the receiver operating characteristic (ROC) curves. The troponin concentration threshold, determined using the ROC curve, identified over half (50.8%) of patients at risk of both death and hospitalization who would otherwise have remained undetected (61 out of 120 patients). Similarly, the troponin ratio identified 53.3% of at-risk patients (64 out of 120 patients) who would not have been recognized using conventional methods (Table 4 and Table 5).

## 3. Discussion

In this prospective study, we found that chronic coronary artery disease patients with elevated troponin levels had a higher risk of death and cardiovascular events, considered as death and HF hospitalization, compared to the patients with lower troponin levels. These findings remained consistent whether troponin was considered as a continuous or categorical variable. Thus, troponin levels may add prognostic information for these patients. In this study, we included consecutive patients with stable CAD, who were compliant with their treatment, and with severe clinical characteristics. They received regular care at an outpatient clinic dedicated to chronic coronary disease management.

Troponin results were normalized, transforming troponin concentrations in a ratio relative to the 99th percentile since our primary interest lies in the range between the detection limit and the 99th percentile, where the influence of gender over the upper reference limit is more pronounced. The ratio provides a common metric for comparing results obtained using different antibody detection kits, as it accounts for the potential discrepancies in antibody-specific epitopes and cutoff values among kits from different manufacturers [12].

We opted to exclude individuals with chronic renal insufficiency as it is acknowledged to have an impact on troponin levels. Conversely, although there is a recognized association between diabetes and troponin levels, we chose to include diabetic patients in the study since they represent more than 50% of the patients under treatment for CAD [2,9]. In addition, no differences were found between the troponin levels of the two groups.

A greater incidence of previous MI and congestive HF was present in the group with higher troponin levels, the third tertile group. Moreover, a higher proportion of women was also observed in this group, even considering the correction for the distinct 99th percentiles that account for gender differences. Interestingly, the results did not show differences across the troponin groups regarding the prevalence of type 2 diabetes mellitus, nor in glucose parameters such as glycemia and glycated hemoglobin. We also did not find differences concerning the number of coronary arteries with obstructive lesions.

Coronary artery disease patients in the third tertile of troponin ratios exhibited two times more risk of overall mortality and combined events compared to the patients in the first tertile. Importantly this association persisted even after multivariate adjustment for important clinical factors. On the other hand, the lack of an association between troponin and the risk of MI and revascularization procedures should be discussed. While these events were infrequent in the present study, their rates were quite consistent across the tertile troponin groups. These results are also in line with what troponin is expected to be related to, as a cardiac muscle protein associated with muscle dysfunction and not with coronary plaque instability. Thus, based on the possible mechanisms involved with its higher blood concentrations, it is plausible that it is associated with events related to cardiac muscle dysfunction and not with cardiac events related to the coronary vessels.

The timing of blood collection varied between patients since they were at different points of their treatment, and the data were consistently gathered upon their initial entry into the protocol. Troponin results suggest that there is a release mechanism that is consistent but does not necessarily imply irreversible cardiac cell necrosis.

The adoption of the new assays of troponin allowed the detection of very low levels of this protein. Several studies were published suggesting that detectable cTn, not necessarily above the 99th percentile, is related to adverse outcomes in chronic cardiac and non-cardiac conditions in the absence of cell necrosis. Some of the studies enrolled populations that were not routinely tested for troponin in an effort to understand the potential prognostic role of troponin and explore the mechanisms underlying its release [13,14,15].

Our findings demonstrated that there is an independent association between troponin levels and the risk of death and combined events over a medium-term follow-up. This suggests that even in well-managed chronic conditions, cardiac tissue may continue to be exposed to stress and vulnerability, potentially resulting in unfavorable outcomes. Various theories attempt to explain the elevation of troponin levels in different clinical situations unrelated to necrosis. Troponin elevation may involve a transient disruption of the plasma membrane with cytoplasmic troponin release, a coronary microvascular dysfunction promoting functional ischemia; the apoptosis of cardiomyocytes, triggered by increased myocardial wall stress; the formation of membrane blebs; imbalances in oxygen supply and demand; inflammation; and increased macromolecule exchange through the membrane due to cardiomyocyte contractions [16,17,18,19].

Blood pressure measurements were not included in this study due to the continuous and controlled follow-up of all patients in the hospital’s outpatient clinics. While this approach ensured stringent control over blood pressure, it can be regarded as a study limitation.

## 4. Materials and Methods

### 4.1. Study Design

This study was a prospective cohort investigation involving consecutive outpatients diagnosed with stable coronary artery disease who underwent treatment at a tertiary hospital between 2020 and 2023. These patients were scheduled for regular appointments with a cardiologist every 6 months as part of their outpatient follow-up.

To be eligible for inclusion, individuals needed to meet the following criteria: be above 18 years of age, have chronic coronary artery disease with luminal stenosis of ≥70%, experience well-controlled clinical symptoms, and be consistently taking prescribed medications. The diagnosis of coronary artery disease was confirmed through angiography that showed at least one vessel branch with a proximal obstructive lesion of 70% of the lumen vessel. All patients were on optimal medical therapy at the time of their entry into the protocol. Optimal medical therapy included the use of aspirin, statins, ACE inhibitors or angiotensin-receptor blockers, and beta-blockers in patients with left ventricular dysfunction or angina. Nitrates and calcium-channel blockers were also used to control angina symptoms.

Patients were excluded from the study if they had experienced an MI within the past 6 months, had ongoing recurrent or unstable angina, or showed clinical or laboratory evidence of renal insufficiency with creatinine levels exceeding 3 mg/dL.

The study was conducted at the Instituto do Coracao (InCor) and was approved on 6 March 2020 by the Ethics Committee of the Hospital das Clinicas HCFMUSP da Faculdade de Medicina da Universidade de Sao Paulo, SP, Brazil (CAAE: 27728619.3.0000.0068).

### 4.2. Laboratory Analyses

Venous blood samples were collected from all patients at the time of their enrollment in the study, regardless of the duration of their disease. High-sensitivity troponin I (hs-cTnI) levels were measured using the ADVIA Centaur TNIH kit (Siemens Healthcare Diagnostics, Tarrytown, NY, USA) on automated equipment from the same manufacturer. The detection limit of the assay is 2.5 ng/L, with intra- and inter-assay coefficients of variation (CV) at 8 ng/L of 6.1% and 8.7%, respectively. The 99th percentile upper reference limit values are 40 ng/L for women and 58 ng/L for men.

For other measurements, venous blood was drawn from fasting patients, typically on or close to the day of their inclusion in the protocol. Serum creatinine, urea, total cholesterol, triglycerides, HDL cholesterol, and glucose levels were determined using commercial colorimetric–enzymatic methods with Dimension EXL equipment from Siemens Healthcare (Newark, DE, USA), along with dedicated reagents. LDL cholesterol (LDL-c) was calculated using the Friedewald equation, or when triglycerides were >300 mg/dL, LDL-c was determined using the LDL direct kit from Siemens on automated equipment. Glycated hemoglobin was assessed in whole blood samples using a turbidimetric method with a specific kit designed for automated Dimension EXL equipment from Siemens Healthcare. Hemoglobin levels were measured with automated equipment, the Abbott Alinity hq analyzer from Abbott Diagnostics, Wiesbaden, Germany. All measurements were performed on the same day of the blood collection.

### 4.3. Clinical Endpoints

Clinical events were tracked and the data on troponin were collected. All clinical endpoints were recorded in data sets and were confirmed based on patient and family information, in case of death. The primary endpoint for analysis included a composite of death from any cause and/or hospitalization for HF. Mortality data were confirmed based on hospitalization records, death certificates, family information, and national death certificates. The diagnosis of MI was based on the patient history of acute coronary syndrome associated with electrocardiographic and laboratory changes that characterize MI. Percutaneous coronary intervention and coronary artery bypass grafting were also investigated and documented. These procedures were indicated if patients evolved with worsening angina symptoms with important physical limitations despite optimal medical therapy that included the use of beta-blockers, nitrates, and calcium-channel blockers in maximal tolerated doses. Revascularization procedures were also indicated in the case of acute coronary syndrome. Patient follow-up was conducted during clinic consultations every 6 months.

### 4.4. Statistical Analysis

The Kolmogorov–Smirnov distribution test was employed to assess the distribution of variables. Values were reported as mean ± standard deviation or median and interquartile range, as appropriate. Categorical data were reported as absolute and relative frequencies.

The troponin results were transformed in the ratio between troponin concentration and the 99th percentile, accounting for gender-related variations, and then categorized into tertiles. Continuous data with symmetrical distribution were compared among the 3 groups using one-way analysis of variance (ANOVA), and when distribution was asymmetrical, the Kruskal–Wallis test. The comparison of categorical data was performed by the chi-square test. Survival data were presented as non-adjusted Kaplan–Meier curves, and the log-rank test was used to compare these data. Multivariate adjustments were calculated by Cox regression models that tested the association between the ratio of cTnI/99th percentile with the occurrence of events, combined and isolated. The predictor was analyzed both as continuous data and also categorized in tertiles. Covariates were included in the regression model based on the differences among them in the baseline analysis (all data with a *p*-value lower than 10% were included in the model), and also if they had a plausible biological association with both the predictor and outcome (such as the severity of coronary disease obstruction and left ventricular ejection fraction). Receiver operating curves were also constructed to assess the optimal cut-off value of troponin I concentration and the ratio of cTnI/99th percentile associated with death and combined endpoints. All tests were two-sided and *p*-values < 0.05 were considered statistically significant. All analyses were performed using R software version 3.5.3 (R Project for Statistical Computing).

## 5. Conclusions

In patients with stable CAD who are adherent to treatment, cTnI/99th percentile is independently associated with combined events, death, and HF hospitalization in a medium-term follow-up.

The study’s outcomes and clinical implications suggest that, unlike the acute phase of myocardial infarction, the persistent release of cTn can lead to lasting clinical implications.

These findings underscore the multifaceted nature of troponin elevation and its potential as a signal of ongoing cell stress and damage, shedding light on the need for further research to elucidate the underlying mechanisms involved.

## Figures and Tables

**Table 1:** Demographic, laboratory, and clinical characteristics of the study population stratified by troponin/99th tertiles.

	Total (*n* = 949)	Group 1 (0.09–0.1) (*n* = 316)	Group 2 (0.14–0.2) (*n* = 316)	Group 3 (0.34–1.12) (*n* = 317)	*p*-Value
Age (years)	67.5 ± 9.5	67.1 ± 9.5	68.0 ± 9.5	67.3 ± 9.5	0.07
Male Sex	660 (69.5%)	249 (78.8%)	215 (68.0%)	196 (61.8%)	<0.001
Hypertension	682 (71.9%)	235 (74.4%)	231 (73.1%)	216 (68.1%)	0.18
Diabetes	494 (52.1%)	163 (51.6%)	167 (52.8%)	164 (51.7%)	0.94
Dyslipidemia	716 (75.4%)	239 (75.6%)	244 (77.2%)	233 (73.5%)	0.55
Previous MI	486 (51.6%)	138 (43.8%)	161 (51.4%)	187 (59.2%)	0.004
Previous Angina	199 (21%)	51 (16.1%)	72 (22.8%)	76 (24.0%)	0.03
Smoking	503 (53.2%)	165 (52.4%)	164 (52.2%)	174 (54.9%)	0.75
Previous PCI	383 (40.5%)	134 (42.4%)	136 (43.3%)	113 (35.6%)	0.16
Previous CABG	332 (35.1%)	115 (36.4%)	116 (36.9%)	101 (31.9%)	0.34
Previous HF	340 (35.9%)	82 (25.9%)	110 (34.9%)	148 (46.7%)	<0.001
LVEF %	55 (40–63)	60 (51–65)	55 (40–61.5)	43 (35–60)	0.49
CAD Single-Vessel Double-Vessel Triple-Vessel	96 (10.6%) 286 (31.5%) 526 (57.9%)	42 (13.7%) 102 (33.2%) 163 (53.1%)	26 (8.7%) 87 (29.1%) 186 (62.2%)	28 (9.3%) 97 (32.1%) 177 (58.6%)	0.12
Creatinine (mg/dL)	1.11 (0.95–1.30)	1.06 (0.9–1.22)	1.12 (0.94–1.31)	1.17 (0.98–1.39)	0.83
Total Cholesterol (mg/dL)	153 (129–188)	149 (126–185)	156 (130–185)	155 (133–192)	0.68
LDL-Cholesterol (mg/dL)	82 (64–109)	77 (62–105)	83 (65–108)	86 (66–113)	0.58
HDL-Cholesterol (mg/dL)	42 (36–50)	42 (35–49)	42 (36–51)	42 (36–49)	0.99
Triglycerides (mg/dL)	120 (87–181)	120 (85–176)	118 (85–179)	126 (90–185)	0.36
Fasting Glucose (mg/dL)	118 (103–149)	119 (105–150)	115 (102–143)	121 (103–152)	0.54
Glycated Hemoglobin (%)	6.5 (5.8–7.7)	6.3 (5.8–7.6)	6.5 (5.9–7.7)	6.6 (5.9–7.9)	0.85
Hemoglobin (g/dL)	14.0 (12.9–15.1)	14.1 (12.9–15.2)	14.0 (13.0–15.1)	13.8 (12.6–14.9)	0.45
Troponin (ng/L)	9.0 (5.0–17.0)	4.0 (3.0–6.0)	9.0 (8.0–11.0)	26.0 (17.0–57.0)	<0.001

Data are presented as median and interquartile range or *n* (%). MI: myocardial infarction; PCI: percutaneous coronary intervention; CABG: coronary artery bypass graft surgery; HF: heart failure; LVEF: left ventricular ejection fraction; CAD: coronary artery disease.

**Table 2:** Incident events in the study population stratified by cTnI/99th tertiles.

	Group 1 (*n* = 316)	Group 2 (*n* = 316)	Group 3 (*n* = 317)	*p*-Value *
HF Hospitalization	4 (1.3%)	13 (4.2%)	25 (8.0%)	<0.001
MI	12 (3.9%)	5 (1.6%)	7 (2.3%)	0.13
PCI or CABG	22 (7.1%)	18 (5.8%)	22 (7.1%)	0.52
Death	19 (6.0%)	28 (8.9%)	41 (12.9%)	0.002
Combined Events (Death + Hospitalization)	22 (7.0%)	40 (12.8%)	58 (18.5%)	<0.001

HF: heart failure; MI: myocardial infarction; PCI: percutaneous coronary intervention; CABG: coronary artery bypass graft surgery. * Cox regression model (univariate analysis).

**Table 3:** Adjusted Cox proportional hazards model for death and combined events (troponin/99th percentile assessed as a categorical variable).

		Group 1 (Reference)	Group 2	Group 3
Overall Death	HR (95% CI), *p*-value	1.0	1.12 (0.60–2.10), *p* = 0.71	2.02 (1.13–3.60), *p* = 0.017
Combined Events	HR (95% CI), *p*-value	1.0	1.33 (0.77–2.32), *p* = 0.31	2.30 (1.37–3.88), *p* = 0.002

HR: hazard ratio; CI: confidence interval. Model adjusted for age, sex, previous MI, angina, heart failure, coronary artery disease, and left ventricular ejection fraction.

**Table 4:** ROC curves of cTnI.

	AUC (95% CI)	Sensibility (%)	Specificity (%)	Cut Off (ng/L)	*p*-Value
Death	0.619 (0.587–0.650)	48.9	69.7	>13	<0.0001
Combined Events	0.645 (0.613–0.675)	50.8	70.8	>13	<0.0001

**Table 5:** ROC curves of cTnI/99th percentile.

	AUC (95% CI)	Sensibility (%)	Specificity (%)	Cut Off (Ratio)	*p*-Value
Death	0.611 (0.579–0.642)	53.4	66.0	0.24	<0.0001
Combined Events	0.639 (0.607–0.669)	53.3	67.7	0.24	<0.0001

## Data Availability

Data is contained within the article.
